# Intraoperative *in vivo* confocal endomicroscopy of the glioma margin: performance assessment of image interpretation by neurosurgeon users

**DOI:** 10.3389/fonc.2024.1389608

**Published:** 2024-05-22

**Authors:** Yuan Xu, Thomas J. On, Irakliy Abramov, Francesco Restelli, Evgenii Belykh, Andrea M. Mathis, Jürgen Schlegel, Ekkehard Hewer, Bianca Pollo, Theoni Maragkou, Karl Quint, Randall W. Porter, Kris A. Smith, Mark C. Preul

**Affiliations:** ^1^ The Loyal and Edith Davis Neurosurgical Research Laboratory, Department of Neurosurgery, Barrow Neurological Institute, St. Joseph’s Hospital and Medical Center, Phoenix, AZ, United States; ^2^ Department of Neurosurgery, Fondazione IRCCS Istituto Neurologico Carlo Besta, Milan, Italy; ^3^ Department of Neurological Surgery, Rutgers New Jersey Medical School, Newark, NJ, United States; ^4^ Department of Neurosurgery, Inselspital, University Hospital of Bern, University of Bern, Bern, Switzerland; ^5^ Department of Neuropathology, Institute of Pathology, TUM School of Medicine and Health, Technical University of Munich, Munich, Germany; ^6^ Institute of Pathology, Lausanne University Hospital, University of Lausanne, Lausanne, Switzerland; ^7^ Neuropathology Unit, Fondazione IRCCS Istituto Neurologico Carlo Besta, Milan, Italy; ^8^ Institute of Tissue Medicine and Pathology, University of Bern, Bern, Switzerland; ^9^ Quint Healthcare, Fürth, Germany

**Keywords:** brain tumor, confocal laser endomicroscopy, glioma, tumor margin, intraoperative imaging, fluorescein sodium

## Abstract

**Objectives:**

Confocal laser endomicroscopy (CLE) is an intraoperative real-time cellular resolution imaging technology that images brain tumor histoarchitecture. Previously, we demonstrated that CLE images may be interpreted by neuropathologists to determine the presence of tumor infiltration at glioma margins. In this study, we assessed neurosurgeons’ ability to interpret CLE images from glioma margins and compared their assessments to those of neuropathologists.

**Methods:**

*In vivo* CLE images acquired at the glioma margins that were previously reviewed by CLE-experienced neuropathologists were interpreted by four CLE-experienced neurosurgeons. A numerical scoring system from 0 to 5 and a dichotomous scoring system based on pathological features were used. Scores from assessments of hematoxylin and eosin (H&E)-stained sections and CLE images by neuropathologists from a previous study were used for comparison. Neurosurgeons’ scores were compared to the H&E findings. The inter-rater agreement and diagnostic performance based on neurosurgeons’ scores were calculated. The concordance between dichotomous and numerical scores was determined.

**Results:**

In all, 4275 images from 56 glioma margin regions of interest (ROIs) were included in the analysis. With the numerical scoring system, the inter-rater agreement for neurosurgeons interpreting CLE images was moderate for all ROIs (mean agreement, 61%), which was significantly better than the inter-rater agreement for the neuropathologists (mean agreement, 48%) (*p* < 0.01). The inter-rater agreement for neurosurgeons using the dichotomous scoring system was 83%. The concordance between the numerical and dichotomous scoring systems was 93%. The overall sensitivity, specificity, positive predictive value, and negative predictive value were 78%, 32%, 62%, and 50%, respectively, using the numerical scoring system and 80%, 27%, 61%, and 48%, respectively, using the dichotomous scoring system. No statistically significant differences in diagnostic performance were found between the neurosurgeons and neuropathologists.

**Conclusion:**

Neurosurgeons’ performance in interpreting CLE images was comparable to that of neuropathologists. These results suggest that CLE could be used as an intraoperative guidance tool with neurosurgeons interpreting the images with or without assistance of the neuropathologists. The dichotomous scoring system is robust yet simple and may streamline rapid, simultaneous interpretation of CLE images during imaging.

## 1 Introduction

Confocal laser endomicroscopy (CLE) is a US Food and Drug Administration–cleared and European CE-marked handheld probe-based cellular-level imaging technology that is used in conjunction with the fluorescent contrast agent fluorescein sodium (FNa) (1). The laser from the probe excites FNa molecules that traverse the damaged blood-brain barrier and remain in the extracellular space of the tissue imaged. The fluorescence emitted by the excited FNa molecules is captured by the probe to generate grayscale images of the microscopic structure of the tissue. In other words, CLE imaging acts effectively as an optical biopsy. This technology has been widely studied and implemented in various surgical and nonsurgical fields and has been recently introduced into neurosurgery (2–4). In surgery for primary invasive brain tumors, determining the presence of tumor infiltration at the margins of resection is challenging despite advancements in surgical imaging technologies (5, 6). The current neurosurgical CLE platform is integrated with a telepathology consultation platform (7, 8). Ever since its initial introduction to clinical use, it has been implemented and evaluated as an intraoperative pathology diagnosis platform.

In our previous study, four neuropathologists who were experienced with CLE imaging reviewed and scored CLE images in a blinded manner that were acquired from glioma margin regions of interest (ROIs) using a proposed six-category numerical scoring system (9). We showed that the numerical scoring system was suitable for reporting and communicating interpretation results, and CLE imaging demonstrated good sensitivity but relatively low specificity at glioma margins.

As this technology begins to gain traction as an intraoperative tool for examining tumor margins, discussion among CLE users brought forth the question of neurosurgeons’ capability to interpret the CLE images. In various instances, neurosurgeons may use CLE intraoperatively without the involvement of a neuropathologist. Unlike neuropathologists, neurosurgeons typically benefit from real-time clinical cues during surgery, such as the appearance of surrounding tissue, brain location from image-guided navigation, exposure to the immediate clinical state of the patient, and performance of previous surgical intervention. This would be particularly interesting from a surgeon’s point of view in that the same person would have the surgical perspective and be able to analyze the pathological results. This is also in line with previous observations that examining CLE images in real time yields better diagnostic performance than storing, reviewing, and interpreting them later (10). It would thus be advantageous for neurosurgeons using CLE to be experienced and effective interpreters of the CLE images they are acquiring.

This study was designed to evaluate whether neurosurgeons experienced with CLE imaging can interpret stored CLE images of glioma margins with accuracy and reliability similar to that of neuropathologists. In addition, we compared the inter-rater reliability and diagnostic performance of the lesional/nonlesional dichotomous scoring system that is commonly used for CLE image interpretation. This study and our earlier study (9) are the first to evaluate the performance of interpreting images of *in vivo* CLE imaging at glioma margins, which is the most important use for real-time intraoperative optical biopsies.

## 2 Methods

### 2.1 Study design

The design of this study mirrors the protocol outlined in our previous study (9), consisting of a multicenter retrospective analysis of CLE images collected from ROIs at glioma margins. The involvement of human participants was reviewed and approved by the Institutional Review Board for Human Research at St. Joseph’s Hospital and Medical Center and the Ethics Commission of the Canton of Bern, Switzerland. The participants provided their written informed consent to participate in this study.

CLE images acquired at glioma margins at Barrow Neurological Institute, St. Joseph’s Hospital and Medical Center, Phoenix, Arizona, and the Inselspital, Bern University Hospital, Bern, Switzerland, were included in this study. FNa was administered at a dose of 5 mg/kg within minutes before CLE imaging for all cases. All CLE images were acquired using either the commercial system (CONVIVO, Carl Zeiss Meditec AG) or the premarket system (Optiscan Five1, Optiscan Imaging Ltd.) that shared the same technology and produced nearly identical images to the commercial system. The tumor margin was defined as the margin of enhancement if the majority of the tumor was enhancing on contrast-enhanced T1-weighted magnetic resonance imaging (MRI) or as the margin of hyperintensity on T2-weighted MRI if the majority of the tumor was not enhancing. The same images used by neuropathologists were used by the neurosurgeons in this study and were assessed in a blinded fashion.

### 2.2 Image interpretation criteria

In the previous study (9), a numerical scoring system was used by neuropathologists to classify ROIs into six categories based on the FNa signal, cellularity, cytological atypia, and histoarchitectural distortion seen in the CLE images (**Supplementary Table 1**). A score of 1 indicated images with the fewest neoplastic features, a score of 5 indicated images with the most neoplastic features, and a score of 0 indicated images characterized by a total lack of any identifiable cellular structure. An “artifact” score was given if none of the CLE images from that ROI were interpretable due to red blood cells or motion artifacts.

For this study, an additional dichotomous scoring system was used (**Table 1**) to assist neurosurgeons in classifying CLE images in an easier and more intuitive way. A score of “Y” for an ROI indicated that tumor infiltration was suspected. A score of “N” for an ROI indicated that the CLE images were insufficient to confirm tumor infiltration. In cases where interpretation of the image was not possible due to presence of motion artifacts or blood, a score of “A” was assigned.

**Table 1:** Proposed dichotomous scoring for confocal laser endomicroscopy image interpretation.

Score	Description	Comments
A	Uninterpretable due to artifacts	Motion artifacts, blood
N	Nonlesional	Insufficient evidence to confirm tumor infiltration
Y	Lesional	Obvious tumor infiltration

### 2.3 Image interpretation and scoring

Anonymized CLE images (single images and image series) and screenshot images of hematoxylin and eosin (H&E)-stained conventional histology sections acquired at the same ROI were uploaded to a cloud database platform (Ambra, Intelerad Medical Systems Inc). Clinical information, including patient age, patient sex, type of tumor, location of the tumor, previous treatment, and contrast enhancement on MRI, was provided for each data set. Four neurosurgeons experienced in CLE imaging and interpretation reviewed the CLE images and assigned a numerical score and a dichotomous score to each ROI based on all the images or image series of that ROI using both scoring systems described above. These neurosurgeons were blinded to the scores assigned by others. Numerical scores assessed by neuropathologists for CLE and H&E images from our previous study using the same protocol were used for comparison (9).

### 2.4 Inter-rater agreement

To evaluate the inter-rater agreement among the four neurosurgeons or four neuropathologists, percent agreement for each ROI was calculated. The mean percent agreement across all ROIs was then calculated. All ROIs were further divided into newly diagnosed tumor, recurrent tumor, low tumor probability (LTP), and high tumor probability (HTP) groups, and percent agreement was calculated for each subcategory. Although neuropathologists used only the numerical scoring system for their evaluations, neurosurgeons used both the numerical and the dichotomous scoring systems. The difference in numerical scoring between the neurosurgeons and neuropathologists was compared using box and whisker plots.

### 2.5 Diagnostic performance of neurosurgeons using CLE

In order to calculate the diagnostic accuracy of the neurosurgeons’ interpretations, the mode of the neuropathologists’ H&E scores for each ROI was converted to a dichotomous lesional/nonlesional notation using the method previously described (9), and this converted score was used as the standard to determine the presence of tumor infiltration. ROIs in which the mode of the H&E scores was ≥3 were considered tumor-positive, while modes <3 were considered tumor-negative. Numerical CLE scores from the neurosurgeons were converted to HTP/LTP notation using the same method as in our previous study (9). The converted numerical CLE scores and the dichotomous CLE scores were compared to the H&E-stained conventional histology standard to calculate diagnostic performance. The sensitivity, specificity, positive predictive value (PPV), and negative predictive value (NPV) of CLE imaging at the glioma margins were calculated using a 2 × 2 table for all ROIs and for the newly diagnosed tumor, recurrent tumor, LTP, and HTP groups. Concordance between the converted CLE score and the dichotomous score was calculated for both individual raters and the overall group.

### 2.6 Statistical analysis

Statistical analysis was performed using IBM SPSS Statistics (version 28.0.1.1, IBM Corp.). A paired *t* test was used to test for statistical significance between neurosurgeons’ and neuropathologists’ inter-rater agreement across ROIs. Ninety-five percent confidence intervals were calculated by multiplying the z-scores and the standard errors for mean agreement, sensitivity, specificity, PPV, and NPV. The Pearson chi-square test was used to compare concordance, sensitivity, specificity, PPV, and NPV between groups. Continuous variables are presented as mean and standard deviation. Categorical variables are presented as number and percentage. A *p* value < 0.05 was considered statistically significant.

## 3 Results

### 3.1 Descriptive analysis

A total of 4275 CLE images from 28 glioma cases that included 56 ROIs were included. Twenty-one cases were from Barrow Neurological Institute and 7 cases were from Bern University Hospital. Of the 28 glioma cases, 11 (39%) were newly diagnosed gliomas (25 ROIs [45%]). The other 17 (61%) were recurrent gliomas (31 ROIs [55%]). The demographic and clinical information of the patients are shown in **Supplementary Table 2**. Thirty-three (59%) ROIs were considered tumor-positive and 23 (41%) were considered tumor-negative based on the interpretation by neuropathologists of the H&E-stained conventional histology sections.

### 3.2 Inter-rater agreement among neurosurgeons and among neuropathologists

**Figure 1:**
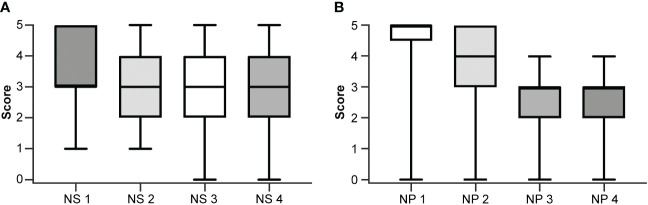
Box and whisker plots showing individual scoring using the numerical scale by **(A)** each neurosurgeon (NS) and **(B)** each neuropathologist (NP). The boxes extend from the 25th to the 75th percentiles. The lines in the middle of the boxes represent the median of the scores and may coincide with the 25th or the 75th percentiles. The whiskers show the maximum and minimum scores given by the rater. *Used with permission from Barrow Neurological Institute, Phoenix, Arizona*.

The inter-rater agreement among the four neuropathologists (48%) was significantly lower than the inter-rater agreement among the four neurosurgeons (61%; *p <*0.01) when using the numerical scoring system to examine glioma margin CLE ROIs. When neurosurgeons used the dichotomous scoring system to examine the same glioma margin CLE ROIs, the inter-rater agreement was 83%. The inter-rater agreements for both scoring systems were individually calculated for the newly diagnosed tumor, recurrent tumor, LTP, and HTP groups (**Table 2**). The inter-rater agreement for neurosurgeons using the dichotomous scoring system was significantly greater than the inter-rater agreement for neurosurgeons using the numerical scoring system (*p* < 0.001) across all ROIs, newly diagnosed glioma, recurrent glioma, LTP, and HTP groups. Differences in numerical scoring between the neurosurgeons and neuropathologists were compared (**Figure 1**).

**Table 2:** Inter-rater agreement among neurosurgeons and among neuropathologists for interpretation of confocal laser endomicroscopy images acquired at glioma margins*.

Category	Agreement among Neurosurgeons	Agreement among Neuropathologists	*p* value†
Numerical scoring system			
Newly diagnosed glioma	59 (52-56)	41 (29-53)	0.03
Recurrent glioma	63 (55-70)	57 (50-64)	0.29
LTP group	55 (49-62)	48 (39-57)	0.26
HTP group	64 (57-71)	48 (40-57)	0.01
All ROIs	61 (55-66)	48 (42-54)	0.007
Dichotomous scoring system			
Newly diagnosed glioma	85 (78-92)		
Recurrent glioma	82 (75-89)		
LTP group	80 (73-88)		
HTP group	86 (79-82)		
All ROIs	83 (79-88)		

HTP, high tumor probability; LTP, low tumor probability; ROIs, regions of interest.

*Data are presented as % (95% confidence interval).

†*p* values under the numerical scoring system category tested the difference in inter-rater reliability between neurosurgeons and neuropathologists for the numerical scoring system.

### 3.3 Concordance between numerical scoring system and dichotomous scoring system

**Figure 2:**
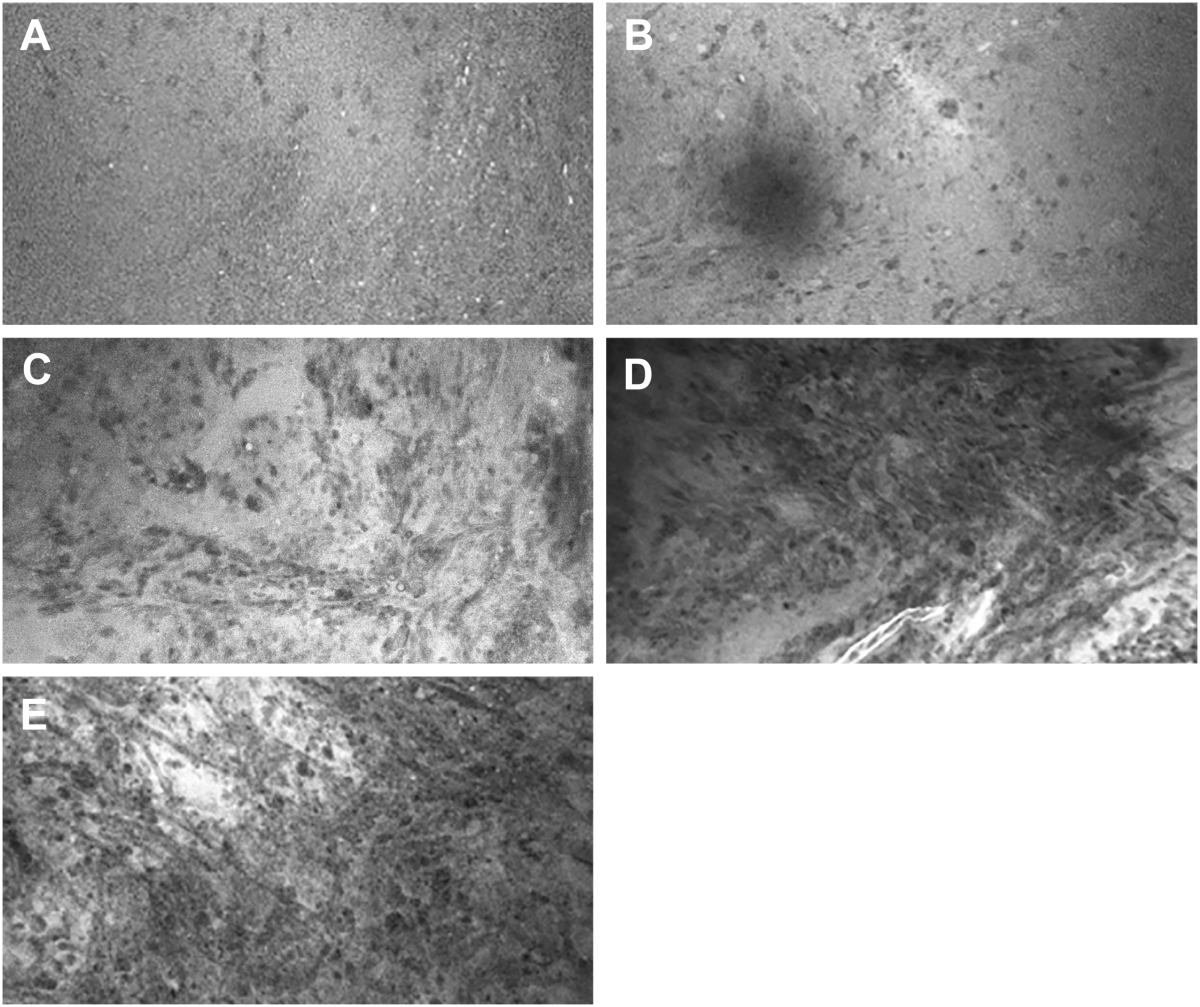
Representative images for CLE image scores in the numerical scoring system. **(A)** Score of 1: CLE image with normal cellularity. **(B)** Score of 2: CLE image with slightly elevated cellularity. This finding is not sufficient to confirm tumor infiltration. **(C)** Score of 3: Significantly elevated cellularity, atypia, or distorted architecture, most likely due to tumor infiltration. **(D)** Score of 4: Marked tumor infiltration is obvious. **(E)** Score of 5: Solid tumor. No ROI was unanimously scored 0, which indicates faint signal and no obvious identifiable structures on the CLE images. *Used with permission from Barrow Neurological Institute, Phoenix, Arizona*.

Four neurosurgeons provided 56 scores for 56 ROIs, using both the numerical scoring system (**Figure 2**) and the dichotomous scoring system. For the numerical scoring system, scores were converted to a dichotomous HTP/LTP notation using the method described previously (9). The converted score from the numerical scoring system was then compared to the dichotomous scoring system to determine concordance between scoring systems for all raters as well as for the newly diagnosed tumor, recurrent tumor, LTP, and HTP groups (**Table 3**). In total, 209 of 224 pairs of rater scores (93%) were concordant. The highest concordance for a rater was 55 of 56 (98%) and the lowest concordance for a rater was 49 of 56 (88%).

**Table 3:** Concordance between dichotomous and numerical scoring systems.

	Concordance (%)	*p* value
Rater		
1	94.5	
2	98.2	
3	92.7	
4	87.5	
Overall	93.3	
Category		
Newly diagnosed glioma	93.0	0.97*
Recurrent glioma	93.5	
LTP group	91.7	0.40†
HTP group	94.5	

HTP, high tumor probability; LTP, low tumor probability.

*p value reflects comparison of concordance for newly diagnosed glioma group to concordance for recurrent glioma group.

†*p* value reflects comparison of concordance for LTP group to concordance for HTP group.

### 3.4 Interpretation of CLE imaging at glioma margins: neurosurgeons vs neuropathologists

For the scores of neurosurgeons using the numerical scale, the overall sensitivity, specificity, PPV, and NPV were 78%, 32%, 62%, and 50%, respectively. For the scores of neuropathologists using the numerical scale, the overall sensitivity, specificity, PPV, and NPV were 79%, 37%, 63%, and 56%, respectively. For the neurosurgeons using the dichotomous scale, the overall sensitivity, specificity, PPV, and NPV were 80%, 27%, 61%, and 48%, respectively. There was no significant difference in diagnostic performance when comparing neurosurgeons and neuropathologists’ ability to interpret CLE imaging. In addition, there was no significant difference in diagnostic performance between the use of the numerical scale and the dichotomous scale by neurosurgeons. The diagnostic performance of CLE imaging at glioma margins for stratified groups is reported in **Table 4**.

**Table 4:** Diagnostic performance of confocal laser endomicroscopy imaging at glioma margins*.

Group	Sensitivity	Specificity	PPV	NPV
Neurosurgeons				
Dichotomous scoring system				
Newly diagnosed glioma	79 (68-90)	27 (13-41)	61 (49-72)	48 (26-69)
Recurrent glioma	81 (71-89)	27 (15-40)	62 (52-72)	48 (29-67)
All	80 (73-87)	27 (18-37)	61 (54-69)	48 (34-62)
Numerical scoring system				
Newly diagnosed glioma	78 (67-89)	30 (16-44)	60 (49-71)	50 (30-70)
Recurrent glioma	77 (68-87)	33 (20-47)	63 (53-73)	50 (33-67)
All	78 (70-85)	32 (22-42)	62 (54-69)	50 (37-63)
Neuropathologists				
Numerical scoring system				
Newly diagnosed glioma	76 (65-88)	48 (32-63)	65 (53-77)	61 (44-78)
Recurrent glioma	80 (71-90)	28 (16-40)	61 (51-71)	50 (31-69)
All	79 (71-86)	37 (27-47)	63 (55-70)	56 (43-69)

NPV, negative predictive value; PPV, positive predictive value.

*Values are presented as % (95% confidence interval).

**Figure 3:**
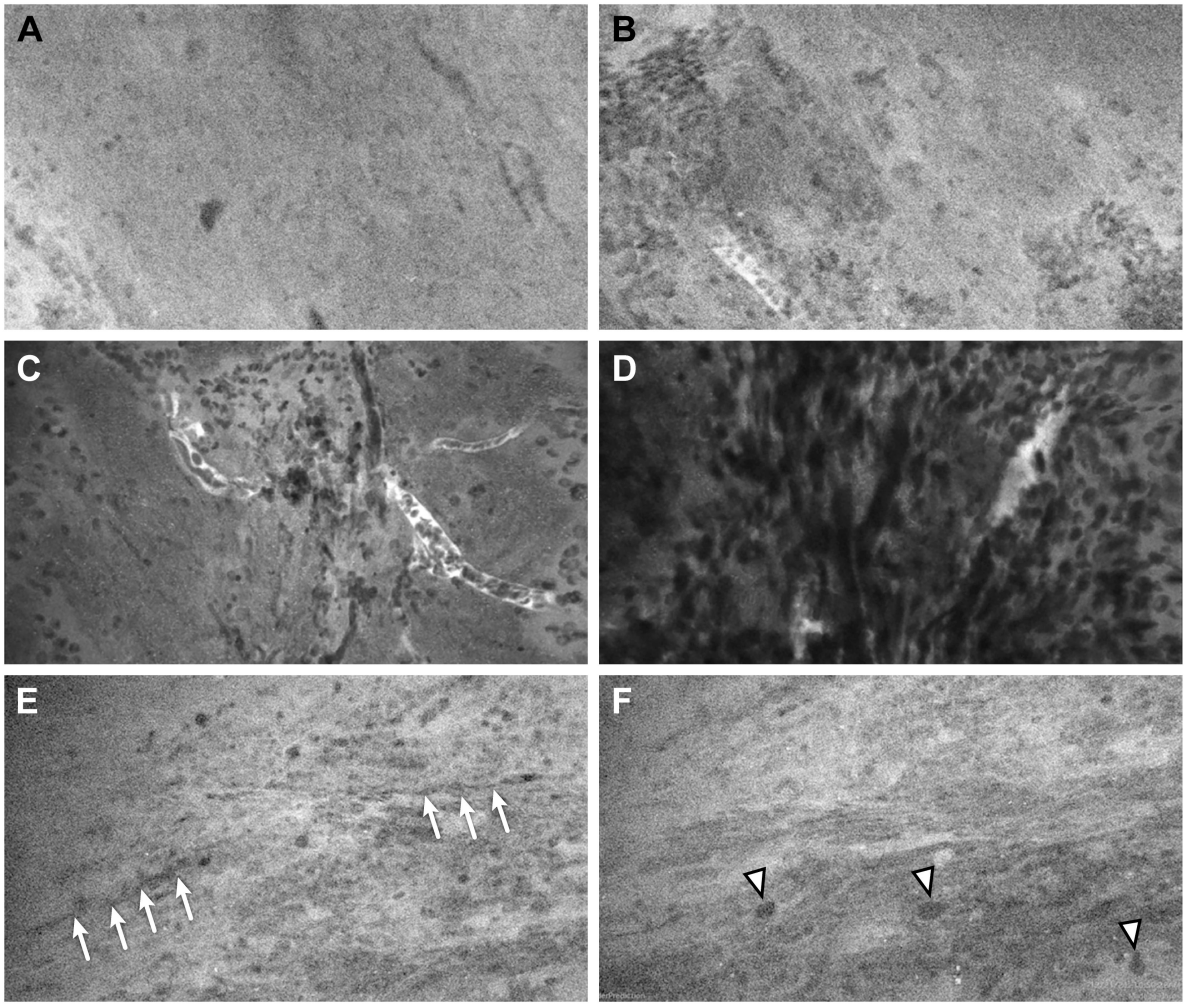
Representative CLE images from 3 ROIs that had a discrepancy of interpretation between neurosurgeons and neuropathologists. In ROI 1, **(A)** one sample showed normal cellularity and no pathological features, suggesting nontumorous tissue, and **(B)** a second sample showed nests of cells that could be interpreted as infiltrating tumor cells or clumps of erythrocytes. In ROI 2, **(C)** one sample showed scattered cells (likely erythrocytes) and several small vessels over a relatively normocellular background and **(D)** a second sample showed highly cellular density, but it was difficult to determine whether the cells represented tumor cells or erythrocytes. In ROI 3, **(E)** one sample showed a relatively normal cellular density and morphology, with cord-like structures (*arrows*) likely representing axons; and **(F)** a second image from the same Z-stack sequence showed some cells with large atypical nuclei (*arrowheads*) that were suspicious for tumor cells. *Used with permission from Barrow Neurological Institute, Phoenix, Arizona*.

Out of the 56 ROIs, 6 (11%) had discrepant interpretations between the neuropathologists and the neurosurgeons after the numerical CLE scores were converted to the dichotomous HTP/LTP notation (**Figure 3**, **Table 5**). Of these, the neurosurgeons’ CLE interpretation matched the tissue biopsy result in three ROIs, and the neuropathologists’ CLE interpretation was consistent with tissue biopsy in the other three.

**Table 5:** Discrepant regions of interest based on the median numerical confocal laser endomicroscopy scores by the neurosurgeons and neuropathologists.

ROI	Neurosurgeons		Neuropathologists		H&E interpretation
	Numerical Score, median	Dichotomous Conversion	Numerical Score, median	Dichotomous Conversion	
1	2	Low tumor probability	3.5	High tumor probability	Nonlesional
2	3	High tumor probability	2.5	Low tumor probability	Lesional
3	2	Low tumor probability	3	High tumor probability	Nonlesional
4	3	High tumor probability	2.5	Low tumor probability	Nonlesional
5	4	High tumor probability	0	Low tumor probability	Nonlesional
6	2.5	Low tumor probability	4	High tumor probability	Lesional

H&E, hematoxylin and eosin-stained histology sections; ROI, region of interest.

## 4 Discussion

In previous *ex vivo* and *in vivo* intraoperative CLE imaging studies where ROIs from both the tumor core and the tumor margin in gliomas were combined, significantly higher sensitivity and specificity in the 90% range were reported (3, 4, 11). We believe this disparity is largely because CLE images acquired at the tumor core in gliomas more frequently show unequivocal diagnostic features such as increased cellularity, obvious nuclear pleomorphism, and atypia, than those acquired at the tumor margin. In this set of two papers, we assessed the capability of CLE imaging specifically at glioma margin ROIs. The low specificity was consistent based on the neurosurgeons’ and neuropathologists’ assessments, indicating an intrinsic limitation of CLE that needs to be substantially improved. In addition, we showed that neurosurgeons who are experienced with CLE imaging can interpret CLE images with similar proficiency compared to neuropathologists. This finding may direct future investigation as an intraoperative guidance tool for brain tumor surgery in addition to an intraoperative diagnostic tool.

### 4.1 Inter-rater agreement among neurosurgeons for CLE image interpretation

The numerical scoring system required users to carefully inspect the CLE images, looking for the presence of specific histopathological features and assessing the extent of tumor infiltration. Although neuropathologists and neurosurgeons usually agreed on the presence or absence of tumor, as reflected in their similar diagnostic performance, the neuropathologists had more disagreement with each other over whether to score a tumor-positive ROI as 3, 4, or 5 or a tumor-negative ROI as 1 or 2. **Figure 1** shows the individual scoring of the four neurosurgeons and the four neuropathologists. The neurosurgeons’ scores spread out more than the neuropathologists’ scores, as indicated by the wider interquartile range. It is worth noting that NS1 and NS2 never assigned “0” to an ROI (suggesting no tumor invasion), while NP3 and NP4 never scored an ROI “5” (suggesting frank tumor invasion). This likely reflected the difference in the neurosurgeons’ and neuropathologists’ approach.

When continuous scanning is being performed, it may not be feasible to scrutinize each image. A dichotomous lesional/nonlesional interpretation based on the overall impression of a series of images is often used. Thus, we also tested the performance of such a dichotomous system compared to the numerical scoring system. When using the dichotomous scale, the inter-rater agreement was excellent (83%). The enhanced inter-rater agreement that was observed for the dichotomous scale can be attributed to its binary nature, which evaluates agreement between just two variables (lesional/nonlesional), in contrast to the numerical scale, which offers a broader range of scoring options, thereby introducing more complexity and potential for variability in ratings. To test whether the dichotomous scoring system is reliable, we converted the numerical scores into the LTP/HTP notation and found a high concordance between the converted scores and the dichotomous scores (93.3% overall, **Table 3**). The high concordance between the dichotomous scoring system and the numerical scoring system showed that the dichotomous scoring system represents an alternative, faster, and simpler way to communicate the CLE imaging results, especially for intraoperative real-time interpretation during scanning.

### 4.2 Diagnostic accuracy of the neurosurgeons’ interpretation

The diagnostic performance calculated from the neurosurgeons’ interpretation was comparable to that of the neuropathologists. Similar to our previous findings, CLE imaging produced higher sensitivity than specificity. No statistical difference was found between the sensitivity, specificity, PPV, and NPV calculated from the dichotomous and numerical scores, indicating that the dichotomous scoring system is sufficient for fast yet reliable reporting of CLE results. After the attempt to lessen the effect of possible CLE-biopsy location mismatch by excluding all ROIs that had discrepancies between H&E scores and CLE scores by both neuropathologists and neurosurgeons, the sensitivity and specificity at glioma margins increased to 80-90% and 45-55%, respectively. The specificity remained low, which likely reflects the limited ability of FNa to identify and distinguish between different cell types encountered at glioma margins. The margin of an invasive glioma is a challenging region because of these variable histological characteristics, especially where tumor cells are found in much less abundance than the tumor core. In addition, surgical microtrauma may be present that disrupts the clear histoarchitecture at the margin.

### 4.3 CLE as a real-time intraoperative guidance system

In this study, four neurosurgeons were presented with the same clinical information as the neuropathologists when interpreting the CLE images. The inter-rater agreement and the diagnostic accuracy can thus be directly compared between the neuropathologists and neurosurgeons. The results from this study suggest that rigorous neuropathology training, including reading H&E-stained histology sections, may not be a prerequisite for CLE image interpretation. Rather, they are able to interpret the images with accuracy that is not inferior to that of their neuropathologist counterparts. This suggests the possibility of CLE being used as a real-time intraoperative guidance tool for neurosurgeons. Neurosurgeons may be able to quickly scan multiple spots within the tumor resection bed, with or without the assistance of a consulting neuropathologist, to search for possible tumor infiltration. In fact, there are experienced neurosurgeon users (author F.R.) who feel comfortable interpreting the images on-the-fly even without input from neuropathologists. Such a method is understandable because it is the neurosurgeon who is operating and controlling the device; thus, it seems that the CLE system is primarily a surgical tool. The noninvasive nature of producing what is essentially a digital optical biopsy would allow the CLE-experienced neurosurgeon to rapidly interrogate a tumor with regionally heterogeneous histoarchitectural characteristics. The learning curve for neurosurgeons to interpret CLE images has yet to be characterized, but based on the feedback from neurosurgeons who actively use CLE, it should not be excessively steep.

Some discussion surrounds whether pathology or neurosurgery benefits more from this technology. CLE has shown good correlation in several studies to frozen and permanent histology (4, 11, 12), and thus, in certain cases, such optical biopsy precludes the need for a physical biopsy, allowing immediate determination of the precise extent of a lesion and facilitating timely intraoperative definitive management. Image processing and display incorporated into the CLE system based on machine learning or artificial intelligence analysis may provide filtering of noninformative or artifactual images and enable standardization of diagnostic features, significantly facilitating the neurosurgeon’s use (13, 14). Nonetheless, this does not obviate the need for neuropathologists’ expertise, especially, for example, where imaging shows unusual histoarchitectural features, in rare tumors, or near or in eloquent cortical regions. Rather, it will improve the quality of interdisciplinary collaboration and the real-time diagnostic process (15). In cases where interpretation is challenging or equivocal, second opinions are always immensely helpful.

### 4.4 Toward cellular imaging-guided precision surgery

FNa-based CLE is currently the only real-time cellular resolution imaging technology approved for neurosurgery, being essentially a hand-held microscope. Such convenient high-resolution intraoperative imaging holds great potential for a transformative period in the precision of brain tumor surgery. Indeed, there is great interest in developing technologies for more precise cancer tumor identification and removal (16).

Improvements in several aspects of the current CLE systems could enhance the reliability of CLE as an intraoperative guidance tool, especially with respect to its application at the glioma margin, where the CLE system is specifically targeted for use. First and foremost, the specificity needs to be improved significantly. Because FNa, the only approved fluorophore for the current CLE systems, is a nonspecific staining fluorophore that does not enter the cells, FNa delineates cell morphology by illuminating the background, making it difficult to discriminate between tumor cells and nonneoplastic cells (eg, normal glial cells, reactive astrocytes, immune cells (17)) that are regularly encountered at the margins of both newly diagnosed and recurrent gliomas. In previous studies, fluorophores such as acriflavine and acridine orange directly stained cell nuclei, whereas cresyl violet stained the cytoplasm of glioma cells in an *ex vivo* setting (18). Fluorophore-biomarker conjugates have also been developed that bind glioma cells specifically for better discrimination of tumor cells (19–21). Implementation of different fluorophores for the CLE probe that directly illuminate tumor cells could markedly increase the specificity of the device.

Another improvement to the current CLE system may be to use it in conjunction with stereotactic navigation. In a previous study (4), we used an image-guided surgical system (S8 Stealth Station, Medtronic, Inc., Dublin, Ireland) to allow precise identification of the CLE probe location, allowing convenient routine registration of the probe with the neuronavigation system, which may increase the accuracy of tumor examination. This setup would allow neurosurgeons to directly visualize the probe position and correlate the CLE ROI to the location on neuroimaging. In essence, the navigation system would serve as a macroscopic roadmap, whereas the CLE would provide microscopic detail. The function of CLE as a digital optical biopsy tool will be increasingly important for inspection of tissue in order to select for detailed molecular or genetic assessment.

Other upgrades to CLE could include the field of view and resolution. The CLE system has inherent limitations in its device capabilities, including the field of view and the frame rate. Currently, magnification in the CLE system is fixed, and the field of view is restricted to 475 μm × 267 μm in the commercial system and 475 μm × 475 μm in the premarket system (11). Diagnostic images with a resolution of 1920 × 1080 pixels are acquired at a scanning speed of 0.75 frames/second. This means that, with an image acquired every 1.3 seconds, the system is prone to motion artifacts due to probe instability and brain pulsation unless the CLE probe is maintained perfectly still and in firm contact with the tissue (22). Increased field of view and scanning speeds, when combined with advanced computer vision and deep learning techniques—covering diagnostic frame detection (13), image style transfer (23), feature localization (14), automatic image classification (24), and other elements—could be integrated into the imaging system to substantially improve image interpretation.

### 4.5 Study limitations

The locations of the CLE image and the tissue biopsy were matched according to the study documentation. Despite the best intention to match the tissue biopsy to the CLE images, there may be slight disagreement due to the lack of a biopsy instrument port built into the probe. Currently, as with many biopsy-imaging correlative studies, neurosurgical instruments are gross in terms of size comparison and the tissue volume removed for study. This may account for the discrepancy between CLE and H&E scores and may have affected the diagnostic accuracy values to some extent. The neurosurgeons were not presented with any clinical information except suspected tumor type, lesion enhancement on MRI, and previous treatment, so we could not assess the effects of this information on the interpretation results. However, we believe that this methodology increases the rigor of the study. As with any other intraoperative diagnostic technology, a learning curve is associated with CLE image acquisition as well as interpretation. For neurosurgeons who have limited histopathology experience, additional training is required to assess microscopic features demonstrated on the CLE images. Due to the retrospective design of this study, we were not able to assess the learning curve.

## 5 Conclusions

Neurosurgeons’ performance in interpreting CLE images of glioma margins was comparable to that of neuropathologists. The inter-rater agreement for neurosurgeons was significantly better using the dichotomous scoring system than using the numerical scoring system. These results suggest that CLE may be used as an intraoperative guidance tool with neurosurgeons interpreting the images with or without assistance from neuropathologists. In addition, a simpler dichotomous scoring system may streamline rapid interpretation of CLE images. CLE technology demonstrates that intraoperative neurosurgical imaging at cellular resolution is possible. However, advancements in more specific fluorophore stains or labels would achieve higher sensitivity and specificity to help identify and discriminate tumor cells. Such advancements could significantly improve the potential for more optimal surgery at the crucial margins of invasive tumors or rapid selection of tumor sites for actual tissue sampling. CLE imaging technology does herald, however, a new era of neurosurgery on a cellular level.

## Data availability statement

The raw data supporting the conclusions of this article will be made available by the authors, without undue reservation.

## Ethics statement

The studies involving humans were approved by the Institutional Review Board for Human Research at St. Joseph’s Hospital and Medical Center and the Ethics Commission of the Canton of Bern, Switzerland. The studies were conducted in accordance with the local legislation and institutional requirements. The participants provided their written informed consent to participate in this study.

## Author contributions

YX: Conceptualization, Data curation, Formal Analysis, Project administration, Writing – original draft, Writing – review & editing. TO: Formal Analysis, Writing – original draft, Writing – review & editing. IA: Data curation, Formal Analysis, Writing – review & editing. FR: Formal Analysis, Writing – review & editing. EB: Formal Analysis, Writing – review & editing. AM: Data curation, Writing – review & editing. JS: Formal Analysis, Writing – review & editing. EH: Data curation, Formal Analysis, Writing – review & editing. BP: Formal Analysis, Writing – review & editing. TM: Formal Analysis, Writing – review & editing. KQ: Conceptualization, Supervision, Writing – review & editing. RP: Data curation, Writing – review & editing. KS: Data curation, Writing – review & editing. MP: Conceptualization, Project administration, Supervision, Writing – original draft, Writing – review & editing.
