# Supplementary material for: Intraoperative in vivo confocal endomicroscopy of the glioma margin: performance assessment of image interpretation by neurosurgeon users
Source: Front Oncol. 2024 May 22;14:1389608. doi: 10.3389/fonc.2024.1389608 (PMC11151089; doi:10.3389/fonc.2024.1389608)
Supplement: Supplementary file 1 [file Table_1.docx]

**Supplementary Material**

**Supplementary Table 1.** Numerical scoring system for image interpretation of confocal laser endomicroscopy and hematoxylin and eosin-stained conventional histology sections

| **Score** | **Description** | **Comments** | **Image Type** | **Group** |
| --- | --- | --- | --- | --- |
| Artifact | Uninterpretable due to artifacts | Motion artifacts, blood | CLE only |  |
| 0 | Faint signal | Suggests absence of tumor infiltration; correlate with intraoperative finding | CLE only | LTP |
| 1 | Normal cellularity | No evidence of tumor; may contain unequivocal reactive change | CLE and H&E | LTP |
| 2 | Slightly elevated cellularity | Insufficient to confirm tumor infiltration | CLE and H&E | LTP |
| 3 | Significantly elevated cellularity, atypia, or distorted architecture | Most likely caused by tumor infiltration | CLE and H&E | HTP |
| 4 | Marked tumor infiltration | Obvious infiltration: <50% of cells are tumor cells | CLE and H&E | HTP |
| 5 | Solid tumor | ≥50% of cells are tumor cells | CLE and H&E | HTP |

Abbreviations: CLE, confocal laser endomicroscopy; LTP, low tumor probability; H&E, hematoxylin and eosin-stained histology sections; HTP, high tumor probability.

**Supplementary Table 2.** Demographic and clinical characteristics of 28 patients with intraoperative in vivo confocal laser endomicroscopy imaging at glioma margins*

| **Characteristic** | **Patients (n = 28)** |
| --- | --- |
| Age, mean (SD), y | 51.3 (14.3) |
| Sex |  |
| Male | 18 (64.3) |
| Female | 10 (35.7) |
| Glioma diagnosis |  |
| Newly diagnosed | 11 (39.3) |
| Recurrent | 17 (60.7) |
| Pathology type |  |
| Astrocytoma | 22 (78.6) |
| Oligodendroglioma | 6 (21.4) |
| Tumor grade |  |
| High grade | 26 (92.9) |
| Low grade | 2 (7.1) |
| Previous treatment (recurrent cases) |  |
| Radiation therapy | 17/17 (100) |
| Chemotherapy | 17/17 (100) |

*Data are presented as number of patients (%) unless otherwise indicated.

From Xu Y, Mathis AM, Pollo B, et al. Intraoperative in vivo confocal laser endomicroscopy imaging at glioma margins: Can we detect tumor infiltration? *J Neurosurg.* 2023:1-10. *Used with permission.*
